# Genomic characterization of a large plasmid containing a *bla*_NDM-1_ gene carried on *Salmonella enterica* serovar Indiana C629 isolate from China

**DOI:** 10.1186/s12879-017-2515-5

**Published:** 2017-07-07

**Authors:** Wei Wang, Zulqarnain Baloch, Zixin Peng, Yujie Hu, Jin Xu, Séamus Fanning, Fengqin Li

**Affiliations:** 1 Key Laboratory of Food Safety Risk Assessment, Ministry of Health, China National Center for food safety Risk Assessment, Beijing, People’s Republic of China; 2 College of Veterinary Medicine, South China Agricultural University, Guangzhou, 510642 People’s Republic of China; 3 UCD-Centre for Food Safety, School of Public Health, Physiotherapy and Sports Science, University College Dublin, Belfield D04 N2E5, Dublin, Ireland; 4 Institute for Global Food Security, School of Biological Sciences, Queen’s University Belfast, Stranmillis Road, Belfast, BT9 5AG Northern Ireland; 5 Microbiology Laboratory, China National Centre for Food Safety Risk Assessment, No.7 Panjiayuan Nanli, Chaoyang District, Beijing, 100021 People’s Republic of China

**Keywords:** Extensively-drug resistance, *Salmonella enterica* serovar Indiana, Carbapenem resistance, *bla*_NDM-1_*gene*, Chicken carcass, China

## Abstract

**Background:**

The *bla*_NDM-1_ gene in *Salmonella* species is mostly reported in clinical cases, but is rarely isolated from red and white meat in China.

**Methods:**

A *Salmonella* Indiana (*S. Indiana*) isolate was cultured from a chicken carcass procured from a slaughterhouse in China. Antimicrobial susceptibility was tested against a panel of agents. Whole-genome sequencing of the isolate was carried out and data was analyzed.

**Results:**

A large plasmid, denoted as plasmid pC629 (210,106 bp), containing a composite cassette, consisting of IS*26*-*bla*_NDM-1_-*ble*_MBL_ *-△trpF-tat-cutA-*IS*CR1-sul1-qacE△1-aadA2-dfrA12-intI1-*IS*26* was identified. The latter locus was physically linked with *bla*_OXA-1_, *bla*_CTX-M-65_, *bla*_TEM-1_-encoding genes. A mercury resistance operon *merACDEPTR* was also identified; it was flanked on the proximal side, among IS*26* element and the distally located on the *bla*_NDM-1_ gene. Plasmid pC629 also contained 21 other antimicrobial resistance-encoding genes, such as *aac(6′)-Ib-cr*, *aac(3)-VI*, *aadA5*, *aph(4)-Ia*, *arr-3*, *blmS*, *brp*, *catB3*, *dfrA17*, *floR*, *fosA*, *mph(A)*, *mphR*, *mrx*, *nimC/nimA*, *oqxA*, *oqxB*, *oqxR*, *rmtB*, *sul1, sul2*. Two virulence genes were also identified on plasmid pC629.

**Conclusion:**

To the best of our knowledge, this is the first report of *bla*_NDM-1_ gene being identified from a plasmid in a *S. Indiana* isolate cultured from chicken carcass in China.

## Background

The New Delhi metallo-beta-lactamase (NDM) is one of the most commonly reported carbapenemase resistance mechanisms in the world [1]. The NDM-1 encoding gene (*bla*_NDM-1_) was first detected in *Klebsiella pneumoniae* recovered from a Swedish patient who was infected with an antibiotic-resistant bacterium acquired in New Delhi, India [2, 3]. Thereafter, this plasmid-mediated NDM-1 resistance mechanism has been widely reported [4]. The presence of *bla*_NDM-1_ was generally associated with resistance to antimicrobial compounds, such as aminoglycosides, beta-lactams and fluoroquinolones. The *bla*_NDM-1_ was found to be located on different large plasmids, which were often readily transferable to other bacterial species [5, 6]. Therefore the dissemination of the *bla*NDM-1-containing plasmids has reduced the therapeutic options available for the treatment of patients [7].

*Salmonella* species is one of the most prevalent zoonotic pathogens that cause outbreaks of gastroenteritis in the world. Recently, many researchers have isolated *Salmonella* from chicken meat (at farm, slaughter house and retail outlets) and its byproducts in China [8, 9]. Moreover, it has been reported that *Salmonella* has the potential to act as a reservoir for different antimicrobial resistance-encoding genes [10]. Of note, transmission of *Salmonella* species from animal to humans via different food chains is well recognized [11]. Currently, NDM-1-producing *Salmonella* species have been reported, but the majority are linked to hospital clinics. However, this resistance mechanism is hardly ever reported in bacteria cultured from the meat of food-producing animals [12–14].

Here, for the first time, we report the isolation of a *S. Indiana* strain recovered from a chicken carcass in China. This bacterium was positive for *bla*_NDM-1_ gene, which was located on a large plasmid, along with several other antimicrobial resistance genes, that conferred an extensively-drug resistance (XDR).

## Methods

### Strain collection and antibiotic susceptibility testing

*S. Indiana* C629 was cultured from a slaughtered chicken carcass sample, in Qingdao, Shandong province, in November 2014. The sample was procured from poultry meat that was ready to dispatch in the local supermarket. The slaughterhouse studied, is the largest slaughter house in Qingdao, Shandong province, with approximately 5000 tons production/month. Antimicrobial susceptibility testing (AST) using the Biofosun® Gram-negative panels (Fosun Diagnostics, Shanghai, China), containing 23 compounds, was carried out using the broth dilution method. The panel of antimicrobial compounds tested included amoxicillin/clavulanic acid, ampicillin, azithromycin, cefazolin, cefepime, cefotaxime, cefoxitin, ceftazidime, ceftiofur, chloramphenicol, ciprofloxacin, colistin, enrofloxacin, florfenicol, gentamicin, imipenem, mequindoxs, meropenem, nalidixic, streptomycin, tetracycline, tigecycline, and trimethoprim/sulfamethoxazole. Data obtained was interpreted according to standards and guidelines described by Clinical and Laboratory Standards Institute (CLSI). Similarly, minimum inhibitory concentration (MIC) values for meropenem were subsequently determined by Etest® (bioMérieux, France).

### Whole genome sequencing, assembly, and annotation

The complete genome of *S. Indiana* C629 was isolated as described by Wang et al. [15]. Briefly, whole-genome sequencing was performed using the Pacific Biosciences RS II platform (SMRT® Pacific Biosciences, Menlo Park, CA, USA). De novo assembly of the reads obtained was carried out using continuous long reads (CLR) following the Hierarchical Genome Assembly Process (HGAP) workflow (PacBioDevNet; Pacific Biosciences) as available in the SMRT® Analysis v2.3 program [16]. The predicted functions of proteins identified were annotated based on homologs when compared to SwissProt (http://www.uniprot.org/uniprot/) clusters of orthologs groups (COG) (http://www.ncbi.nlm.nih.gov/COG/), and the NCBI Prokaryotic Genome Annotation Pipeline (PGAP) based on the Best-placed reference protein set and GeneMarkS+. The complete genome of *S. Indiana* plasmid pC629 was deposited in the NCBI database with the fallowing accession number CP015725 (plasmid pC629).

### Sequence analysis of the plasmid pC629 genome

Antimicrobial resistance genes in the genome were predicted using the Comprehensive Antibiotic Resistance Database (CARD) (https://card.mcmaster.ca/analyze) [17]. Virulence factor database (VFDB) (http://www.mgc.ac.cn/VFs/main.htm) was used to predict the presence of virulence factors in the genome of plasmid pC629 [18]. Finally, the antibacterial Biocide and Metal Resistance Genes Database (BacMet) (http://bacmet.biomedicine.gu.se/ ) was used to predict the presence of any metal-resistance genes contained in the genome of plasmid pC629 [19].

## Results

### Susceptibility to a panel of antimicrobial compounds

In this study, it was found that *S. Indiana* C629 isolate was only susceptible to colistin (MIC 0.5 mg/L) and resistant to 18 compounds representing 10 different antimicrobial classes **(**Table 1 **)**. Of note the *S. Indiana* C629 isolate was found to be resistant to imipenem (MIC 4 mg/L) and meropenem (MIC 8 mg/L), both belong to carbapenem antibiotics. Although there are no standards available for antimicrobial agents of mequindox (MIC >64 mg/L), streptomycin (MIC >32 mg/L), ceftiofur (MIC >32 mg/L) and tigecycline (MIC <0.5 mg/L) in the CLSI guideline, MIC measurements for these antimicrobials have shown that the activity of these antimicrobial agents may also be compromised when being considered either for treatment of infection cases or growth promotion applications **(**Table 1 **)**.

**Table 1:** Antimicrobial susceptibility of *S. Indiana* C629 to a panel of 23 antimicrobial agents^a^

Class	Antimicrobial Agent	MIC (mg/L)	R/I/S
Penicillins	Ampicillin (AMP)	>128	R
Cephalosporins	Cefepime (FEP)	>16	R
	Cefoxitin (CFX)	>32	R
	Cefazolin (CFZ)	>32	R
	Cefotaxime (CTX)	>16	R
	Ceftazidime (CAZ)	>32	R
	Ceftiofur (TIO)^c^	>32	-^b^
Carbapenems	Imipenem (IMI)	4	R
	Meropenem (MEM)	8	R
beta-lactamase inhibitor combinations	Amoxicillin/Clavulanic Acid (AMC)	>64/32	R
Polymyxin	Colistin (CT)^c^	0.5	S
Folate pathway inhibitors	Trimethoprim/sulfamethoxazole (SXT)	>8/152	R
Aminoglicosides	Streptomycin (STR)	>32	-^b^
	Gentamicin (GEN)	>32	R
Tetracyclines	Tetracycline (TET)	>32	R
	Tigecycline (TGC)	<0.5	-^b^
Phenicols	Chloramphenicol (CHL)	>32	R
	Florfenicol (FFC)^c^	>16	R
Macrolides	Azithromycin (AMZ)^c^	>64	R
Fluoroquinolones	Nalidixic (NAL)	>128	R
	Ciprofloxacin (CIP)	>4	R
	Enrofloxacin (ENO)^c^	>8	R
Mequindox	Mequindoxs (MEQ)^c^	>64	-^b^

^a^: Interpreted according to the CLSI.^b^There is no interpretative standard for this compound. ^c^Used as a feed additive in animal production in China.

### Analysis of the genome to identify antimicrobial resistance-encoding genes

Whole genome of plasmid pC629 was circular. The total size of plasmid pC629 was determined to be 210,106-bp. The average GC content was 48.6% and it was predicted to encode 223 open reading frames (ORFs) which covered almost 83.4% of the structure. The CARD database was queried to identify resistance related genotypes in the genome of plasmid pC629.

A total of 30 antimicrobial resistance genes (two genes were identified as *sul1*) were identified and which encoded resistance to 19 antimicrobials. The resistance-encoding region in plasmid pC629 was bracketed by several IS*26* elements that were located in different orientations.

### Analysis of the genome to identify virulence-encoding genotypes

**Fig. 1:**
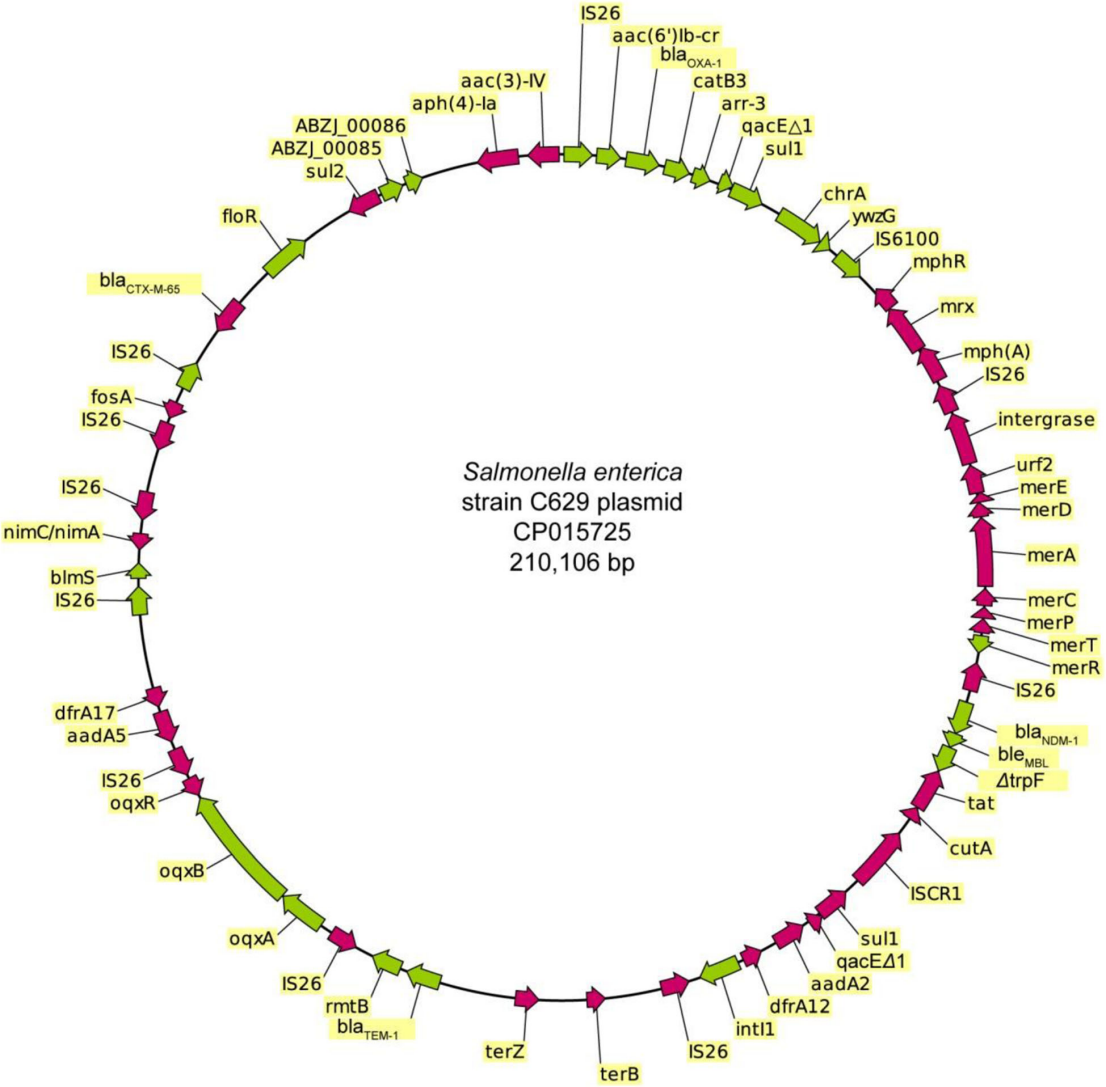
Representation of plasmid pC629. Antibiotic resistance genes, metal resistance genes, and virulence factor genes were shown in the circle

To identify potential virulence genes in the plasmid pC629 genome, the virulence factors listed in the Virulence Factors Database (VFDB) were aligned to the ORF protein sequences using BLASTP and filtered with 50% identity and 90% match length. By using this approach, two virulence genes, such as dDE_Tnp_1, matched *abzi_00085* and *abzi_00086,* were found on plasmid pC629 (Fig. 1).

### Analysis of the genome to identify metal resistance-encoding genotypes

In this study we used the antibacterial biocide and metal resistance genes database (BacMet) to predict the presence of antibacterial biocides and metal-resistance genes on the genome of plasmid pC629. These results predicted the presence of mercury and tellurium resistance genes on plasmid pC629.

## Discussion

To the best of these authors knowledge, we report on the successful isolation and characterization of the metallo-beta-lactamase encoding *bla*_NDM-1_ gene harboring *S. Indiana* strain, cultured from a chicken carcass in China. This bacterium expressed a diverse resistant phenotype. In addition to its antimicrobial resistance-encoding gene repertoire, this bacterium also possessed several virulence genes along with various metal-encoding resistance genes. Generally, the poultry production operation demonstrated good pathogen control and reduction strategies; however, the isolation of *Salmonella* from slaughtered meat that has already been passed by processing checks is very striking. Interestingly, all of these resistant antimicrobial agents tested in this study are widely used at human and veterinary clinics.

**Fig. 2:**
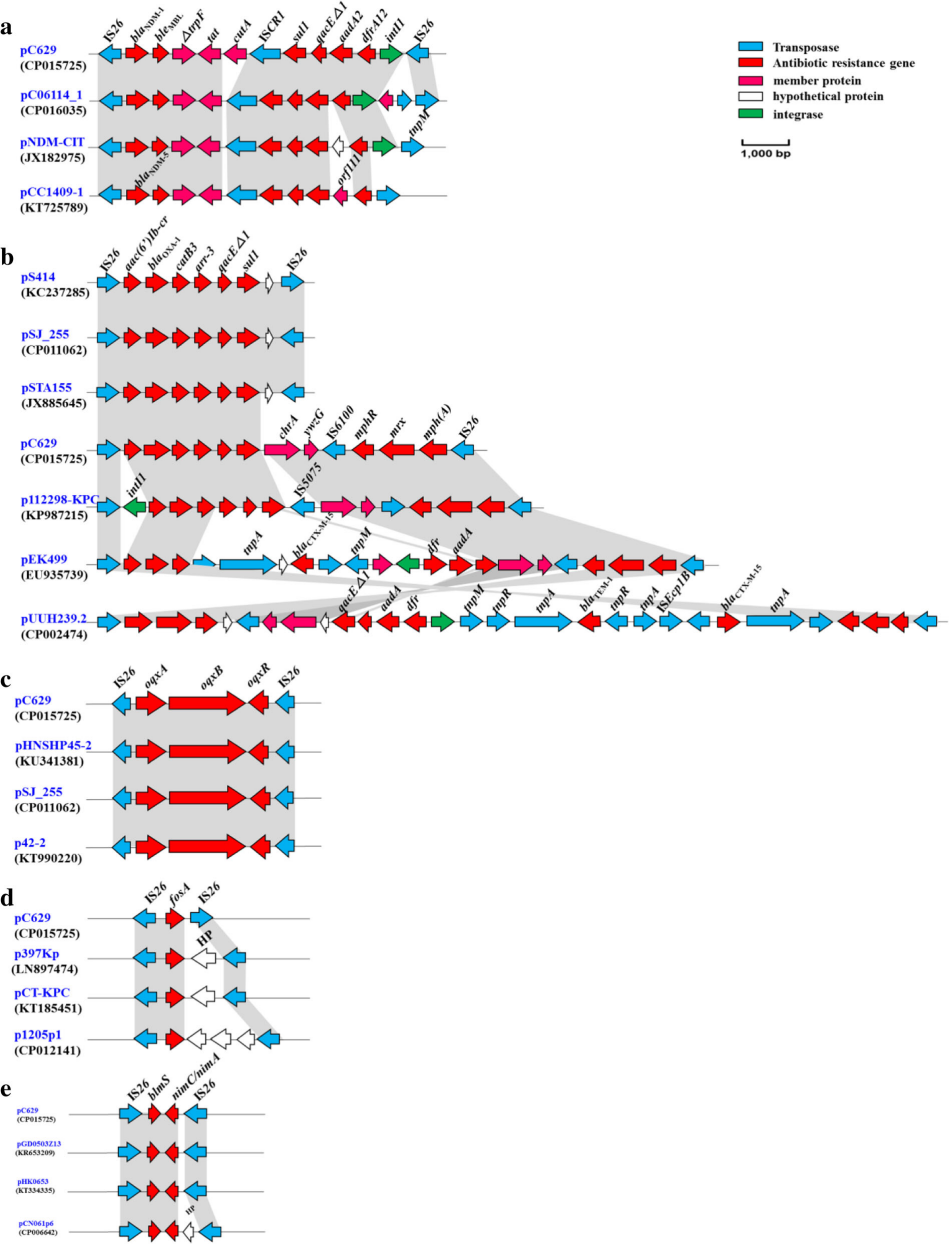
Major structural features of plasmid pC629 compared with several plasmids from NCBI. **a**. Major structural features of plasmid pC629 compared with plasmids pC06114_1, pNDM-CIT, and pCC1409-1 identified in *Escherichia coli*, *Citrobacter freundii*, and *Klebsiella pneumoniae*, respectively. ORFs are shown as arrowheads (blue arrows, IS*26*, IS*CR1*, and *tnpM*; red arrows, antibiotic resistance genes; pink arrows, membrane protein, MP; white arrows, hypothetical proteins, HP; green arrows, integrase related genes –see the key to the right hand side of the panel). **b**. Major structural features of plasmid pC629 compared with plasmids pS414, pSJ_255, pSTA155, p112298-KPC, pEK499, and pUUH239.2 identified in *Salmonella* Indiana, Escherichia coli, *Salmonella* Indiana, *Citrobacter freundii*, *Escherichia coli* and *Klebsiella pneumoniae*, respectively. ORFs are shown with arrowheads (blue arrows, IS*26*, IS*6100* and other transposase related genes; red arrows, antibiotic resistance genes; pink arrows, membrane protein, MP; white arrows, hypothetical proteins, HP; green arrows, integrase related genes –see the key to the right hand side of the panel). **c**. Major structural features of plasmid pC629 plasmid compared with plasmids pHNSHP45-2, pSJ_255, and p42-2 all identified in *Escherichia coli*. ORFs are shown with arrows (blue arrows, IS*26*; red arrows, antibiotic resistance genes –see the key to the right hand side of the panel). **d**. Major structural features of plasmid pC629 compared with plasmids p397Kp, pCT-KPC, and p1205p1 identified in *Klebsiella pneumoniae*, *Klebsiella pneumoniae*, and *Shigella flexneri*, respectively. ORFs are shown with arrows (blue arrows, IS*26*; red arrows, antibiotic resistance genes; white arrows, hypothetical proteins, HP –see the key to the right hand side of the panel). **e**. Major structural features of plasmid pC629 plasmid compared with plasmids pGD0503Z13, pHK0653, and pCN061p6 identified in *Escherichia coli*, *Salmonella* species and *Escherichia coli*, respectively. ORFs are shown with arrows (blue arrows, IS*26*; red arrows, antibiotic resistance genes; white arrows, hypothetical proteins, HP –see the key to the right hand side of the panel)

The *bla*_NDM-1_ gene was embedded in an IS*CR1* complex class 1 integron of 11.8-kb (Figs. 1 and 2). This region was bracketed by two IS*26* elements that were positioned in the same orientation. These observations suggested that the gene may have been acquired as a composite transposon. Similar structures had also been reported in *E. coli* (CP016035) and *Citrobacter freundii* (JX182975) (Fig. 2) [20, 21]. Interestingly, *Klebsiella pneumoniae* (KT725789) also harbored a *bla*_NDM-5_, that is located on plasmid pCC1409–1 (Fig. 2) and whose structural arrangement aligns with our observation here [22]. Downstream of the *bla*_NDM-1_, a *ble*_MBL_ gene is located which encodes resistance to bleomycin and this is followed by a truncated phosphoribosyl anthranilate isomerase gene (denoted as *△trpF*), a twin-arginine translocation pathway signal protein gene (encoded by *tat*) and a dihydroorotate dehydrogenase gene (*cutA*). Further downstream, an IS*CR1* element was identified which was located proximally to a class 1 integron; in addition to a gene cassette containing *dfrA12-aadA2* in the classical *heal-to-tail* arrangement. The similar genetic arrangement have also been reported in an *E. coli* (CP016035), in which a complex class 1 integron harboring an IS*CR1* element was identified [23]. The insertion elements IS*CR1* and IS*26* are known to mediate the mobilization of *bla*_NDM-1_ in Enterobacteriaceae and non-Enterobacteriaceae species [21, 24]. Moreover, the *intI1* gene was followed by a copy of insertion element IS*26* located on the distal side. A similar genetic arrangement has also been reported in pNDM-CIT (JX182975) [21].

In addition to the *bla*_NDM-1_, plasmid pC629 also contained an additional multidrug resistance cassette known as a Bush class 2 beta-lactam resistance-encoding gene *bla*_OXA-1_, which was closely located to the NDM-1 containing composite transposon (Figs. 1 and 2). The *bla*_OXA-1_ gene was preceded by a copy of insertion element IS*26*, and *aac(6′)Ib-cr*, followed by c*atB3*, *arr3*, *qacE△1*, and s*ul1*. Remarkably, the *bla*_OXA-1_ gene associated with IS*26* insertion had also been detected in NDM-negative Enterobacteriaceae, including an *E. coli* containing plasmid pSJ_255 (CP011062, unpublished), a *Citrobacter freundii* 112,298 plasmid p112298-KPC (KP987215), two plasmids pS414 (KC237285) and pSTA155 (JX885645) from *S. Indiana*, respectively [25, 26]. Moreover, several other resistance genes encoded on plasmids pS414 and pSTA155 have been reported which are colocated on the IncHI2 plasmid. It has already been suggested that these genes are associated with a multidrug resistant in *S. Indiana* isolated in China [26]. While, in the downstream region of these resistance genes a chromate transporter protein (encoded by *chrA*) and a putative DNA-binding protein (*ywzG*) were identified. However, this locus was structured differently in the plasmids pSJ_255, pS414, and pSTA155 compared to plasmid pC629.

In this study, we also identified an interesting resistance gene locus consisting of IS*6100-mphR*-*mrx*-*mph(A)*-IS*26*, wherein the *mphR, mrx, and mph(A)* genes encoded different macrolide 2′-phosphotransferases [27–29]. This locus has already been reported in three OXA-encoding plasmids, such as p112298-KPC (KP987215) from *Citrobacter freundii*, pEK499 (EU935739) from *E. coli*, and pUUH239.2 (CP002474) from *Klebsiella pneumonia* [26, 30, 31]. The orientation of the IS*6100* element contained in plasmid p112298-KPC opposes that in plasmid pC629 (Fig. 2). Additionally, the locus IS*6100*-*mphR*-*mrx*-*mph(A)*-IS*26* has already been reported ad it has been suggested that this locus can facilitate the transmission of these resistance mechanisms to other bacteria [32].

In this study, we identified a *oqxRAB* operon on plasmid pC629 and this locus was flanked by IS*26* elements (Figs. 1 and 2). This gene confers low level resistance to fluoroquinolones. Additionally, this gene has 100% sequence similarity at the nucleotide level with other structures identified in in *E. coli* such as plasmids pHNSHP45–2 (KU341381) [33]. pSJ_255 (CP011062, unpublished), and p42–2 (KT990220, unpublished). Furthermore, it has also been reported that transconjugants carrying *oqxA* and *oqxB* exhibited a 4- to 16-fold high MIC to (fluoro) quinolones, and a 16- to 64-fold high MIC against quinoxalines [34]. A fosfomycin resistance encoding gene *fosA* flanked with IS*26* (Fig. 2) and further it was associated with *bla*_CTX-M-65_. We suspected that *bla*_CTX-M-65_ may play role in the maintenance and dissemination of *fosA* gene. Moreover, both *fosA* and *bla*_CTX-M-65_ were separated by only six genes as shown in Fig. 1. Therefore, we suggest that these genes may be transmitting together [35].

In this study, we also identified the bleomycin resistance encoding gene *blmS* along with the 5-nitroimidazole-based (5-Ni) antimicrobial resistance-encoding genes *nimC*/*nimA* on plasmid pC629, which were flanked by inverted IS*26* elements (Fig. 2). A similar conserved sequence has already been reported on plasmids in *E. coli* GDZ13, including plasmid pGD0503Z13 (KR653209, unpublished), in *S.* Typhimurium ST06–53 on plasmid pHK0653 (KT334335, unpublished), and in *E. coli* PCN061 on plasmid pCN061p6 (CP006642) [36]. Furthermore, these isolate also contained 9 other antimicrobial resistance-encoding genes which we also found on plasmid pC629. These genes encode resistant to various antimicrobials, and are, *aac(3)-IV*, *aadA5*, *aph(4)-Ia*, *rmtB* all of which encode rsistance to aminoglycosides*; brp*, encoding resistant to bleomycin; *dfrA17*, encoding resistance to trimethoprim*; floR*, encode resistance to florfenicol; *sul2*, resistance to sulphonamides and *bla*_TEM-1_ the classical resistance gene encoding resistance to beta-lactam compounds.

Interestingly, we found that all of the multi-drug resistance clusters identified on plasmid pC629 and all of these multi-drug resistance clusters were flanked by IS*26.* On the basis of this genetic structure we suggested that pC629 has the potential to disseminate antibiotic resistance genes to other species and the host. In this study we also found two virulence genes named as dDE_Tnp_1, matched *abzi_00085* and *abzi_00086* on plasmid pC629 (Fig. 1), both have already been characterized in *Acinetobacter baumannii* MDR-ZJ06 which is a multidrug-resistant bacterium detected and isolated from patient in China [37]. It has already been suggested that these two genes were participated in composition of the capsule gene cluster, which plays an important role in protecting bacteria from the host innate immune response [38].

**Fig. 3:**
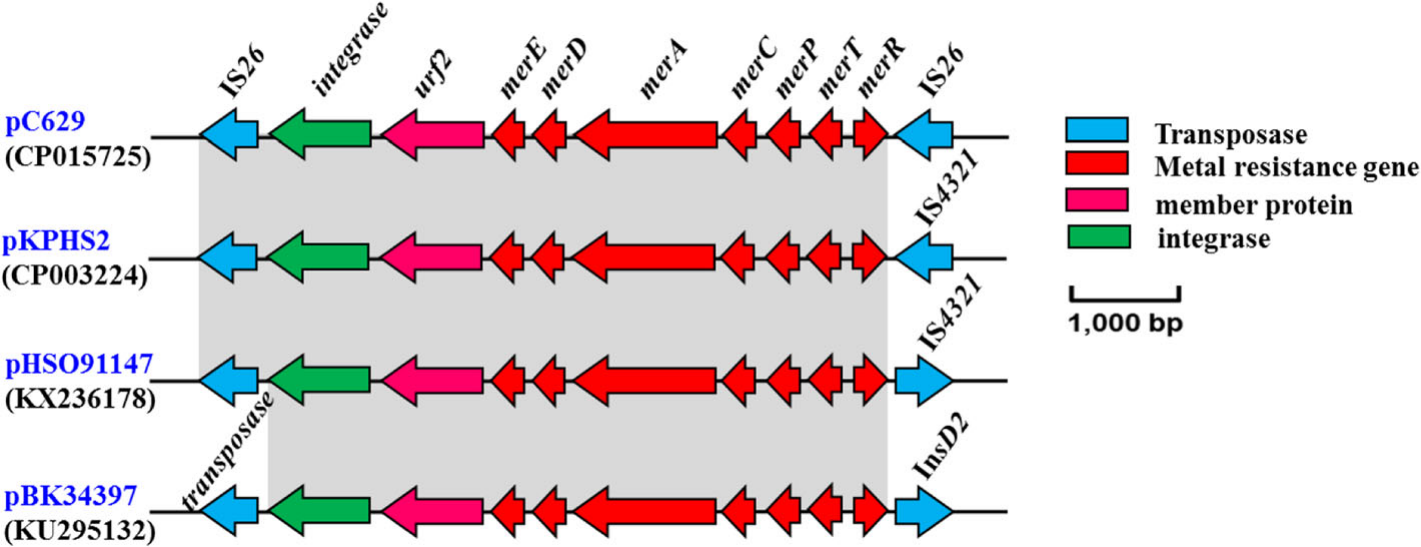
Major structural features of plasmid pC629 compared with plasmids pHSO91147, pBK34397, and pKPHS2 identified in *Klebsiella pneumoniae*, *Escherichia coli* and *Klebsiella pneumoniae*, respectively. ORFs are shown with arrows (blue arrows, transposase; red arrows, metal resistance genes; pink arrows, membrane protein, MP; green arrows, integrase related genes -–see the key to the right hand side of the panel)

Our results predicted the existence of mercury and tellurium resistance gene on plasmid pC629 genome (Fig. 1). Later, we identified 7 mercury resistance genes, and all of these mercury resistance genes flanked by IS*26* elements (IS*26*-*int*-*urf2*-*merE*-*merD*-*merA*-*merC*-*merP*-*merT*-*merR*-IS*26*) (Fig. 3). Similar physical map has also been reported on plasmids pHSO91147 (KX236178, unpublished); pBK34397 (KU295132, unpublished), and pKPHS2 (CP003224) [39], with some insertion sequence variations. Levels of mercury have been elevated in the environment due to industrial pollution. Therefore, many bacterial species have developed detoxification strategies to combat its deleterious effects [40]. The gene *merA* is one of the components contained within the *mer* operon, and this is often associated with mobile genetic elements, including transposable elements and plasmid [41]. Therefore, mercury resistance is transferable among bacterial species via horizontal gene transfer (HGT) mechanisms. Several researchers have suggested that the *mer* operon is physically linked with one or more antimicrobial resistance-encoding genes [42]. In this study, the mercury resistance genes were found to be linked to the *bla*_NDM-1_ gene as follows, IS*26*-*int*-*urf2*-*merE*-*merD*-*merA*-*merC*-*merP*-*merT*-*merR*-IS*26*-*bla*_NDM-1_-*ble*_*MBL*_ *-△trpF-tat-cutA-*IS*CR1-sul1-qacE△1-aadA2-dfrA12-intI1-*IS*26*. This observation provides further evidence of the potential role of *mer* genes in the dissemination of resistance genes.

## Conclusion

To the best of our knowledge, this is the first report describing the characterization of a large XDR expressing plasmid with the metallo-beta-lactamase encoding blaNDM-1 gene cultured from a *S. Indiana* strain isolated from chicken carcass in China. Several multi-drug resistance gene clusters were identified and flanked by IS26 elements. Metal-encoding genes and various metal-encoding resistance determinants were also identified. These data could be used proactively to assist the poultry industry in China to develop food safety measures, designed to limit the transmission of these XDR bacteria and other biological hazards from food-producing animals.

## Abbreviations

VFDB: Virulence factor database
CARD: Comprehensive Antibiotic Resistance Database
COG: clusters of orthologs groups
HGAP: Hierarchical Genome Assembly Process
CLR: Continuous long reads
AST: Antimicrobial susceptibility testing
CLSI: Clinical and Laboratory Standards Institute
MIC: minimum inhibitory concentration
